# 11.1 EFFECT OF INTERVAL TRAINING ON METABOLIC RISK FACTORS IN OVERWEIGHT INDIVIDUALS WITH PSYCHOSIS: A RANDOMIZED CONTROLLED TRIAL

**DOI:** 10.1093/schbul/sby014.039

**Published:** 2018-04-01

**Authors:** Ahmed Jérôme Romain, Cédine Fankam, Antony Karelis, Elaine Letendre, Gladys Mikolajacks, Emmanuel Stip, Amal Abdel-Baki

**Affiliations:** 1 University of Montreal Hospital Research Centre; 2 University of Quebec at Montreal; 3 University Hospital of Montreal

## Abstract

**Background:**

Among adults with psychotic disorders, negative symptoms as unhealthy lifestyle habits contribute to a high prevalence of metabolic syndrome and obesity. Lifestyle interventions, mainly physical activity (PA) has emerged as an essential component. Furthermore, interval training (IT) was found to be efficacious in other populations but poorly studied among people with psychosis.

The objective was to determine the effects of a 6-month IT program on metabolic, anthropometric, and psychiatric/functional outcomes.

**Methods:**

Randomized controlled trial comparing the effects of a bi-weekly 30 minutes supervised IT program to a waiting list of overweight individuals with psychosis. Body composition and metabolic risk factors (blood pressure, insulin resistance, lipid profile) were measured at baseline and every 3 months. The groups were compared on an intent to treat basis with repeated-measures mixed linear models with the restricted maximum of likelihood method of estimation.

**Results:**

Sixty-seven individuals (28 control: waiting list; 39 IT intervention) with psychosis (60.6% men, mean age: 31.0 ± 7.2 years old; BMI: 32.0 ± 6.1 kg/m^2^, waist circumference: 107.7 ± 13.3 cm) were included in the study, and 67.2% completed the study. Attendance for the IT sessions was 61.8% and the dropout rate was 32%. IT was associated with significant improvements on waist circumference (-2.72 cm, SE = 1.34; p = 0.04), negative symptoms (-2.93, SE = 1.34; p = 0.03), social (SOFAS) (+5.23, SE = 2.39; p = 0.03) and global functioning (+7.34, SE = 2.05; p < 0.001). The effects of exercise in the first-episode psychosis (FEP) sub-group were similar to those of the entire cohort.

**Discussion:**

These promising results suggest that IT may be used as a treatment strategy for the management of metabolic complications and possibly improve social functioning in obese individuals with psychotic disorders. Further studies are needed to understand if IT could prevent weight gain and metabolic complications if used before these comorbidities emerge and to understand factors associated with the persistence of exercising.

